# Metabolic subtypes and immune landscapes in esophageal squamous cell carcinoma: prognostic implications and potential for personalized therapies

**DOI:** 10.1186/s12885-024-11890-x

**Published:** 2024-02-19

**Authors:** Xiao-wan Yu, Pei-wei She, Fang-chuan Chen, Ya-yu Chen, Shuang Zhou, Xi-min Wang, Xiao-rong Lin, Qiao-ling Liu, Zhi-jun Huang, Yu Qiu

**Affiliations:** 1 Clinical Laboratory Department, The Second Affiliated Hospital of Fujian Medical University, 362000 Quanzhou, Fujian P. R. China; 2 Shengli Clinical Medical College, Fujian Medical University, Fuzhou, Fujian 350001 P. R. China; 3 Center for Experimental Research in Clinical Medicine, Fujian Provincial Hospital, 350001 Fuzhou, Fujian P. R. China; 4 Stomatology Department, The Second Affiliated Hospital of Fujian Medical University, 362000 Quanzhou, Fujian P. R. China; 5 Central Laboratory, The Second Affiliated Hospital of Fujian Medical University, 362000 Quanzhou, Fujian P. R. China; 6 Esophageal Surgery Department, The Second Affiliated Hospital of Fujian Medical University, 362000 Quanzhou, Fujian P. R. China; 7 Reproductive Center, The Second Affiliated Hospital of Fujian Medical University, 362000 Quanzhou, Fujian P. R. China

**Keywords:** Tumor immune microenvironment, Esophageal carcinoma, Prognosis assessment, Personalized treatment, MTHFD2

## Abstract

**Background:**

This study aimed to identify metabolic subtypes in ESCA, explore their relationship with immune landscapes, and establish a metabolic index for accurate prognosis assessment.

**Methods:**

Clinical, SNP, and RNA-seq data were collected from 80 ESCA patients from the TCGA database and RNA-seq data from the GSE19417 dataset. Metabolic genes associated with overall survival (OS) and progression-free survival (PFS) were selected, and k-means clustering was performed. Immune-related pathways, immune infiltration, and response to immunotherapy were predicted using bioinformatic algorithms. Weighted gene co-expression network analysis (WGCNA) was conducted to identify metabolic genes associated with co-expression modules. Lastly, cell culture and functional analysis were performed using patient tissue samples and ESCA cell lines to verify the identified genes and their roles.

**Results:**

Molecular subtypes were identified based on the expression profiles of metabolic genes, and univariate survival analysis revealed 163 metabolic genes associated with ESCA prognosis. Consensus clustering analysis classified ESCA samples into three distinct subtypes, with MC1 showing the poorest prognosis and MC3 having the best prognosis. The subtypes also exhibited significant differences in immune cell infiltration, with MC3 showing the highest scores. Additionally, the MC3 subtype demonstrated the poorest response to immunotherapy, while the MC1 subtype was the most sensitive. WGCNA analysis identified gene modules associated with the metabolic index, with SLC5A1, NT5DC4, and MTHFD2 emerging as prognostic markers. Gene and protein expression analysis validated the upregulation of MTHFD2 in ESCA. MTHFD2 promotes the progression of ESCA and may be a potential therapeutic target for ESCA.

**Conclusion:**

The established metabolic index and identified metabolic genes offer potential for prognostic assessment and personalized therapeutic interventions for ESCA, underscoring the importance of targeting metabolism-immune interactions in ESCA. MTHFD2 promotes the progression of ESCA and may be a potential therapeutic target for ESCA.

**Supplementary Information:**

The online version contains supplementary material available at 10.1186/s12885-024-11890-x.

## Introduction

Esophageal carcinoma (ESCA) is the 7th most commonly diagnosed cancer worldwide [1], and those with advanced ESCA still suffer from limited treatment options and poor survival rates [2]. First-line chemotherapy regimens for advanced ESCA typically involve fluorouracil- or platinum-based treatments, which achieve a response rate of 40–60% [3]. However, if first-line treatment fails, patients have a median overall survival (OS) of only 5–10 months, and there is currently no efficient standard second-line therapy available. Despite the comprehensive characterization of the human genome [4, 5], targeted therapy for ESCA remains challenging. The tumor–node–metastasis (TNM) classification system is widely used in clinics to assess risk and make treatment decisions [6]. However, even cases with similar clinicopathological characteristics can exhibit significantly different risks of death and recurrence due to substantial molecular heterogeneities [7, 8]. Therefore, it is crucial to further investigate the molecular mechanisms associated with the genetic diversity of ESCA to enable precise diagnosis and the development of individualized treatments.

Whole-genome mRNA expression profiling refers to the comprehensive analysis of gene expression patterns in the entire genome of cancer cells, providing detailed information about the molecular characteristics and functional changes in tumors [9]. By analyzing the expression levels of thousands of genes, researchers can identify gene signatures, pathways and molecular subtypes associated with specific tumor phenotypes or patient outcomes to improve our understanding of tumor biology and identify novel therapeutic targets [10–12]. Thus, while the TNM staging system remains essential for clinical decision-making in esophageal carcinoma, whole-genome mRNA expression profiling research offers additional layers of molecular information that can potentially enhance personalized medicine approaches, improve prognostic accuracy, guide treatment selection, and facilitate the discovery of novel therapeutic targets [13]. However, the relationship between these identified genes and the clinical characteristics of ESCA still requires comprehensive elucidation, especially considering the lack of validation of these identified targets in cell lines and patient samples.

Cancer has been proposed as a disease associated with metabolic disturbances [14]. Various genes and mutations linked to cancer interfere with different metabolic processes that support the proliferation of cancer cells, including aerobic glycolysis, one-carbon metabolism, and glycogenolysis [15, 16]. In ESCA, intratumoral metabolism is also influenced by the heterogeneity of gene mutations [17, 18]. Glycolytic ESCA cells experience a state of relative oxidative stress due to increased levels of reactive oxygen species (ROS), which is counterbalanced by the activation of redox metabolic pathways [19]. Additionally, metabolic changes such as obesity and elevated triglycerides (TG) observed in metabolic syndrome (MetS) are associated with the risk [20, 21]. Furthermore, a retrospective study has confirmed that MetS, characterized by hypertriglyceridemia and impaired fasting glucose, predicts a higher risk of relapse in early ESCA cases [22]. Numerous studies have suggested that immune function can undergo plasticity in different metabolic contexts [23, 24]. Some research focuses on specific metabolic patterns to modulate immune polarization and function, opening possibilities for treating immune-related diseases like cancer [25]. Previous studies have also demonstrated the impact of the tumor microenvironment (TME) on T cell metabolism, particularly in terms of tumor response, offering new insights for opportunities to enhance T cell response through metabolic manipulation, potentially improving the effectiveness of anticancer immunotherapy [26]. Overall, these findings emphasize the importance of investigating the genetic landscape of ESCA at the metabolic level. Thus, accurate identification and characterization of metabolic subpopulations are crucial for a better understanding of ESCA and optimizing anticancer immunotherapy's effectiveness.

In this study, we aimed to investigate the importance of metabolism-immune interactions and contribute to the understanding of ESCA biology. For this, we classify ESCA patients into metabolic subtypes based on gene expression profiles from the GSE19417 and TCGA-ESCA datasets, investigate the relationship between these subtypes and immune landscapes, establish a metabolic index for accurate prognosis assessment, identify metabolic genes associated with the index, validate their expression in patient samples and cell lines, and assess their functional significance. Collectively, the findings provide important insights into ESCA heterogeneity, which could be used as a reference for strategizing personalized treatment interventions for ESCA patients.

## Materials and methods

### ESCA datasets

To investigate the characteristic changes of ESCA in metabolism-related pathways, we collected clinical, SNP, and RNA-seq data from 80 ESCA patients from the TCGA database (https://cancergenome.nih.gov/) based on the following criteria: (a) availability of comprehensive follow-up information and (b) availability of comprehensive gene expression profiles in ESCA. Furthermore, we obtained the expression levels and clinical data from the GSE19417 dataset (*n* = 70, samples with insufficient prognosis data were excluded) from the GEO database [27].

### Data preprocessing

The clinical data of the ESCA samples obtained from TCGA were successfully matched with the corresponding RNA-seq data. Additionally, the ENSG identifiers were mapped to GeneSymbol, allowing us to obtain expression profiles for 21,652 genes. Similarly, for the samples from the GSE19417 dataset, the clinical data were consistent with the RNA-seq data, and expression profiles for 20,101 genes were obtained by mapping chip probes to gene names using probe annotation files.

### Metabolic ESCA subtype identification

In this study, a total of 2,923 metabolic genes enriched in 117 metabolic pathways were obtained from the Molecular Signatures Database (MSigDB) [28]. These genes were then used to select metabolic genes associated with survival based on a threshold of log-rank *P* < 0.05. Among these, expression data from 163 metabolic genes associated with survival were used for k-means unsupervised clustering. The clustering process was performed using the "ConsensusClusterPlus" package [29] with 500 iterations. To determine the optimal cluster number, curves were plotted based on the consensus score and cumulative distribution function (CDF). Subsequently, SigClust analysis was conducted to compare the two subtypes and assess the significance of the clustering results.

### Prediction of activity of immune-related pathway, immune infiltration, as well as response to immunotherapy

In this study, the ESTIMATE algorithm was employed to calculate the ESTIMATE/immune/stromal scores and tumor purity for all cancer samples [30]. The xCell algorithm [31] was used to predict the relative levels of different types of human immune cells within the tumor microenvironment (TME). To validate the results, established approaches such as EPIC [32] and MCPcounter [33] were combined. The activities of immune pathways in various subtypes were predicted using Single-sample GSEA (ssGSEA) with the GSVA R package [34]. Enrichment scores were used to indicate the extent of up- or downregulation of coordinated genes in individual samples. The response to immunotherapy was assessed using the tumor immune dysfunction and exclusion (TIDE) algorithm (http://tide.dfci.harvard.edu/). A higher TIDE score suggests a higher likelihood of immune escape, indicating that patients are less likely to benefit from immunotherapy.

### Establishment of the typical metabolic index for ESCA cases

To establish a typical index for classifying metabolic subtypes, linear discriminant analysis (LDA) was employed. A total of 163 metabolic genes associated with prognosis were utilized, and each segment was subjected to Z-transformation. Fisher's LDA optimization criterion was used to ensure maximal dispersion of the centroids for each group. As a result, a linear combination, denoted as A, was determined to maximize the inter-class variance relative to the intra-class variance. The model indicated that the initial two features were sufficient to distinctly differentiate the different subtypes.

### Weighted gene co-expression network analysis (WGCNA) and clustering analysis

Gene transcription data were analyzed using Weighted Gene Co-expression Network Analysis (WGCNA) [35] to identify metabolic genes associated with co-expression modules. The gene expression data were collected from the TCGA database, and specific parameters were set as follows: a cluster threshold of 5, a median absolute deviation greater than 5%, and a β value of 50%. The expression matrix was then transformed into a topological matrix. Modules were obtained using average linkage with the following parameters: a height of 0.3, a minimum module size of 30, and a depth split of 1.

### Sample collection and preparation

Twenty-seven patients diagnosed with esophageal squamous cell carcinoma (ESCA) were included in this study. The patient inclusion criteria were: (1) treatment naïve patients, (2) underwent surgical resection, (3) pathologically confirmed as ESCA, and (4) had complete baseline data required for data analysis for this study. Those who did not consent to the anonymous use of their tissue samples, underwent previous cancer treatment and were synchronously diagnosed with other cancer were excluded. The patients received treatment at the Department of Gastrointestinal Surgery of Fujian Medical University Second Affiliated Hospital. Prior to their participation, written informed consent was obtained from all patients, which was approved by the Institutional Review Board of Fujian Medical University Second Affiliated Hospital (2021 − 414). The detailed clinical characteristics of the patients are presented in Supplementary Table 1.

During surgery, fresh esophageal carcinoma and adjacent normal tissue samples were cryopreserved and stored in liquid nitrogen. Before the experiment, the specimens were thawed at room temperature.

### Cell culture

The human normal esophageal epithelial Het-1A cells and esophageal squamous cell carcinoma cells (TE-1) were purchased from Shanghai Cell Resource Center (Shanghai, China). The cells were cultured in RPMI-1640 (Wako, Osaka, Japan) containing 10% fetal bovine serum (FBS) and antibiotics (100 U/ml penicillin and 100 µg/ml streptomycin) in a humidified 5% CO_2_ incubator at 37°C.

### Small interfering RNA (siRNA) experiment

TE-1 cells were selected to investigate the functional role of MTHFD2. The experiment included two groups: the siNC Group and the si-MTHFD2 Group. The siRNAs for MTHFD2 (si-MTHFD sense 5’-GAGCAGUUGAAGAAACAUATT-3’, si-MTHFD antisense 5’-UAUGUUUCUUCAACUGCUCTT-3’) and the siRNAs for negative control (si-NC sense 5’-UUCUCCGAACGUGUCACGUTT-3', si-NC antisense 5’-ACGUGACACGUUCGGAGAATT-3’) were purchased from GenePharma (Shanghai, China).

TE-1 cells were inoculated into 6-well plates with a density of 1.5 × 10^5^ cells per well. Transfection mixtures of si-MTHFD2 and siNC were added to the cells, respectively, according to the manufacturer's instructions (Thermo Fisher Scientific), at a final concentration of 30 nM/well. 48 h after transfection, the transfection effect was detected by qRT-PCR and western blot.

### Gene expression analysis by real-time qPCR

RNA was extracted using TRIzol protocol. Next, cDNA synthesis was performed using the PrimeScript RT reagent Kit (Perfect Real Time) following the provided instructions. Reverse transcription of RNA into cDNA was carried out using reverse transcriptase enzyme. The expression levels of target genes, namely SLC5A1, MTHFD2, and NT5DC4, were determined using real-time PCR with the TB Green® Premix Ex Taq fluorescent II kit (Tli RNaseH Plus). The reaction mixture was prepared according to the manufacturer's instructions, and PCR amplification was carried out using a Roche LightCycler 480II amplification instrument. The reaction conditions included an initial denaturation step at 95°C for 30 seconds, followed by 40 cycles of denaturation at 95°C for 5 seconds and annealing/extension at 60°C for 30 seconds. The primer sequence is shown in Supplementary Table 2.

### Western blot

The cells were lysed using a protein lysis buffer supplemented with protease inhibitors, and the protein concentration was determined using a protein assay kit. Equal amounts of protein were separated by sodium dodecyl sulfate-polyacrylamide gel electrophoresis (SDS-PAGE). The proteins were then transferred onto PVDF membrane, followed by blocking using a blocking buffer to prevent non-specific binding and incubated with the primary antibody against MTHFD2 (ab151447, abcam, UK) overnight at 4°C. After washing, the membrane was incubated with a secondary antibody conjugated with horseradish peroxidase (HRP) for one hour at room temperature. Protein bands were visualized using an enhanced chemiluminescence (ECL) substrate, and the signals were captured using a chemiluminescence imaging system. The protein expression level of MTHFD2 was normalized to a loading control GAPDH.

### Cell counting Kit-8 (CCK-8) assay

Briefly, the transfected TE-1 cells were inoculated in a 96-well plate with approximately 5,000 cells per well and incubated under appropriate conditions for 24 hours. Then, the 10 µL of CCK-8 reagent was added to each well according to the manufacturer's instructions, and the cells were further incubated at 37°C for 1 h, the absorbance value of each well at 450 nm was detected using a microplate analyzer (Multiskan EX, Lab systems, Helsinki, Finland). Each experiment was repeated thrice.

### Colony formation assay

After transfection, the cells were plated in six-well plates at a density of 1000 cells per well and incubated in RPMI-1640 medium supplemented with 10% FBS at 37°C and allowed to grow undisturbed for 14 days to view the visible colonies formed by each cell group. After the incubation period, the colonies were fixed with 4% paraformaldehyde to preserve their structure, stained with 0.2% crystal violet (Sigma), and photographed using an appropriate imaging system. Lastly, the number of cell colonies was recorded for both the siNC group and the si-MTHFD2 group. Each experiment was repeated thrice.

### Transwell assay

Matrigel (BD Biosciences) was dissolved overnight at 4°C and then diluted in complete medium at a ratio of 1:3. Around 50 µL of the diluted Matrigel mixture was added to the upper and lower compartments of each well in a 24-well plate (Millipore). The plate was then placed in a 37°C cell incubator for 30 minutes to allow the Matrigel to coagulate. Next, The cells were suspended in 200 µL serum-free medium and added to the upper compartment of the Transwell insert, and 600 µL whole medium was added to the lower cavity. After incubation for 24 hours, the matrix and upper compartment cells were carefully removed. Cells invading and settling in the submembrane cavity through matrix coating are stained with crystal violet. Images of the stained cells were captured using a Nikon frontal microscope. Ten random fields of view were selected, and the number of invaded cells was counted in each field. Each experiment was repeated thrice.

### Statistical analysis

R software (version 3.5.3, http://www.R-project.org) was used for statistical analysis. The relationship between clinical features and different ESCA subtypes was determined using the chi-squared test or Fisher's exact test. Correlation coefficients were determined using Pearson's correlation and distance correlation analyses. The contingency table was checked using a two-sided Fisher exact evaluation. In addition, Kaplan-Meier evaluation was employed to generate cluster survival curves, whereas statistical differences were analyzed using the log-rank test. In multiple testing, FDR correction was performed to reduce the false-positive rate (FPR). All the basic research experiments were replicated at least three times. The data are expressed as mean ± standard deviation, and the means of two groups were determined using the Wilcoxon test for outliers and unpaired Student's t-test for normally distributed variables. One-way ANOVA and Kruskal-Wallis test were used separately for parametric and nonparametric approaches for comparison between different groups. A difference of *P* < 0.05 (two-tailed) was considered statistically significant.

## Results

### Molecular subtypes identified based on metabolic genes

**Fig. 1:**
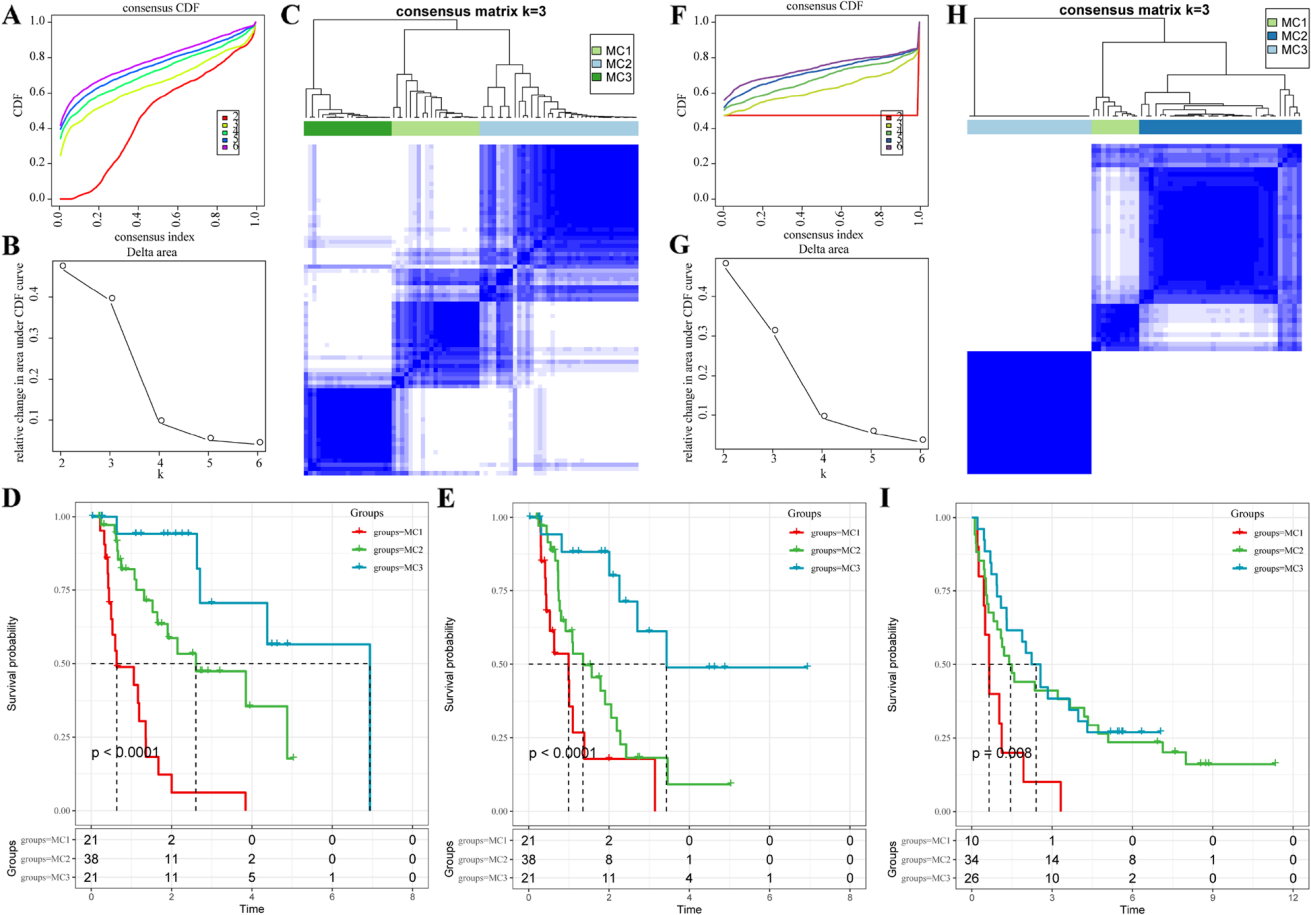
Identification of metabolic subtypes in the ESCA sample from the TCGA and GEO cohorts. **A** The cumulative distribution function (CDF) curves of consensus scores based on different subtype number (k = 2 ~ 6) in the TCGA-ESCA cohort. **B** The CDF Delta area curve of all samples in the TCGA-ESCA cohort when k = 3. **C** The consensus score matrix of ESCA samples in the TCGA-ESCA cohort when k = 3 (1 = MC1, 2 = MC2, 3 = MC3). **D** and **E** Kaplan–Meier curves showing the distinct OS (D) and PFS (**E**) of patients in the three metabolic-related subtypes in the TCGA-ESCA cohort. **F** The cumulative distribution function (CDF) curves of consensus scores based on different subtype number (k = 2 ~ 6) in the GEO-ESCA cohort. **G** The CDF Delta area curve of all samples in the GEO-ESCA cohort when k = 3. **H** The consensus score matrix of samples in the GEO-ESCA cohort when k = 3 (1 = MC1, 2 = MC2, 3 = MC3). **I** Kaplan–Meier curves showing the distinct OS of patients in the three metabolic-related subtypes in the GEO-ESCA cohort

In this study, the ESCA subtype database was obtained from MsigDB based on the expression profiles of metabolic genes. Univariate survival analysis revealed that of the 117 metabolic pathways involving 2,923 genes, 163 were associated with TCGA-ESCA sample prognosis. As a result, these 163 metabolic genes were selected for further analysis using an unsupervised consensus algorithm. Using ConsensusClusterPlus, the cancer tissues from each dataset were classified into k distinct subtypes (k = 2, 3, 4, 5, and 6). Based on the Cumulative Distribution Function (CDF) curves of the consensus scores, the optimal classification was achieved with k = 3 for both the TCGA-ESCA and GEO datasets (Fig. 1A and 1F). SigClust analysis confirmed diverse consensus clusters (k = 3) compared to pairwise comparisons (Fig. 1B-C and G**-**H). Prognosis analysis of the three metabolic phenotypes in the GEO and TCGA databases demonstrated significant differences, with MC1 having the poorest prognosis and MC3 having the best prognosis (Fig. 1D-E and I). Additionally, clinical characteristics, including TNM stage, stage, grade, age, and sex, were analyzed across the different subtypes. The results showed no significant differences in the distribution of these clinical characteristics between the subtypes in both the TCGA-ESCA and GEO datasets (Supplementary Fig. 1). These findings indicate that the classification of ESCA samples based on metabolic genes exhibits high reproducibility across diverse datasets, enabling effective stratification of patient prognosis.

### Metabolic profiles of diverse subtypes

**Fig. 2:**
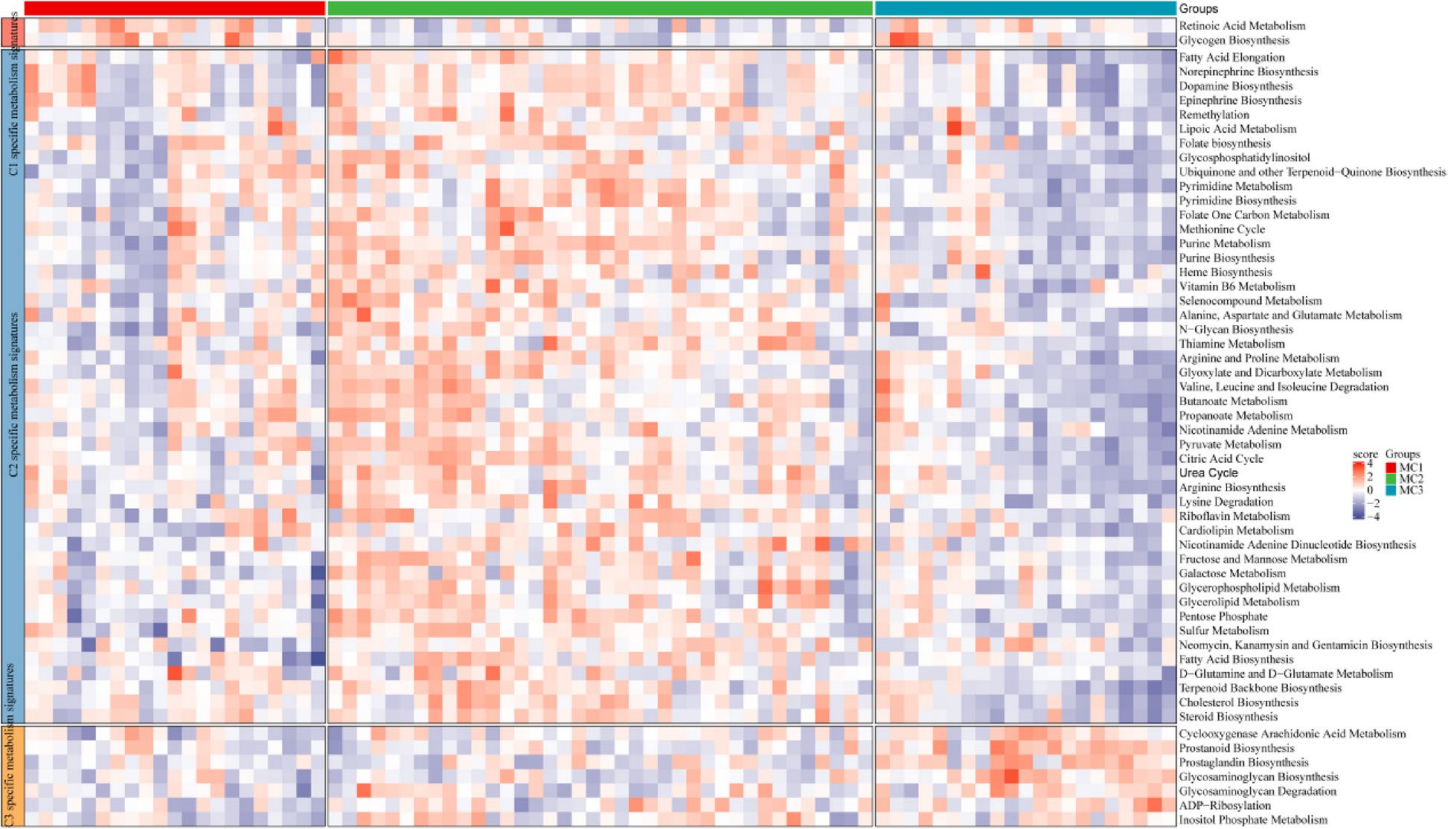
Metabolic profiles of diverse subtypes

In this study, GSVA was employed to investigate the heterogeneity in metabolic pathways among the three subgroups of ESCA. Figure 2 displays the results, indicating the enrichment of specific metabolic pathways in each subtype. The MC1 subtype, associated with the poorest prognosis, exhibited enrichment in two metabolic pathways, namely retinoic acid metabolism and glycogen biosynthesis. On the other hand, the MC3 subtype, associated with the best prognosis, showed enrichment in seven metabolic pathways, including glycosaminoglycan metabolism, prostanoid biosynthesis, prostaglandin biosynthesis, and ADP-ribosylation. The MC2 subtype (*n* = 42) mainly demonstrated enrichment in pathways such as the citric acid cycle, pentose phosphate pathway, and fatty acid metabolism. These findings highlight the differential activation of metabolic pathways across diverse subtypes, underscoring the genetic significance of classification based on metabolic genes.

### ESCA immunogenicity

**Fig. 3:**
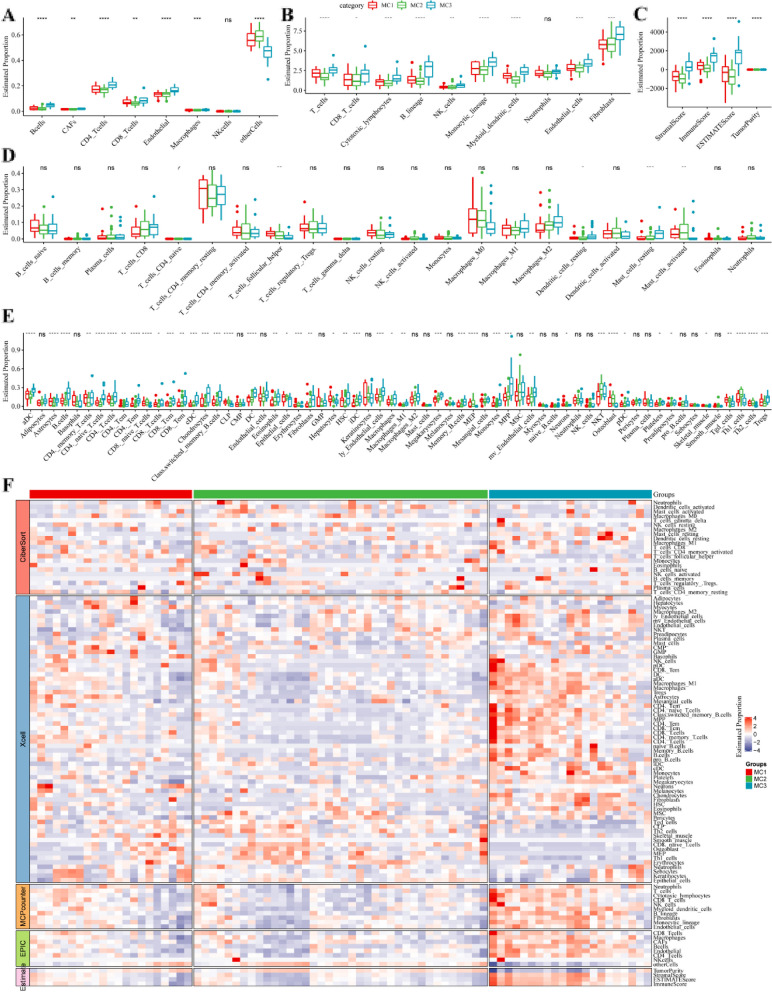
Immune profiles for the three metabolic subtypes in the TCGA-ESCA cohort. **A** The levels of immune infiltrating cells in the samples from three ESCA subtypes obtained by the EPIC algorithm. **B** The levels of immune infiltrating cells obtained by MCPcounter. **C** The immune scores, stromal scores and ESTIMATE scores of the samples from three ESCA subtypes. **D** Proportion of immune cell components calculated by CIBERSORT software. **E** The levels of immune infiltrating cells obtained by the xCELL approach. **E** The heatmap showed the immune infiltration levels in samples from three subtypes obtained by diverse scoring algorithm

In the TCGA dataset we used five software programs, EPIC, MCPcounter, ESTIMATE, CIBERSORT, and xCell, to evaluate the immune cell infiltration. In TCGA-ESCA samples, the proportions of B cells, CD8_Tcells, and Endothelia immune cells were significantly higher in MC3 than in MC1 and MC2 in the EPIC approach (Fig. 3A). Further analysis using the MCP counter approach revealed significant differences in immune cell infiltration within the tumor microenvironment (TME) across the subgroups. The CM3 subgroup exhibited the highest scores for most immune cell types, while the MC1 subtype had relatively lower scores, and the MC2 subtype had the lowest scores (Fig. 3B). Similar results were obtained using ESTIMATE score (Fig. 3C). The software calculations of CIBERSORT allowed us to observe that most of the immune cells were not significant between metabolic subtypes (Fig. 3D). In addition, most of the 64 immune cells assessed by xCell were found in higher proportions in MC3 than in MC1 and MC2 (Fig. 3E). By evaluating the five immune infiltration software, we found significant differences in immune characteristics between subgroups (Fig. 3F), with MC3 having the highest immune infiltration.

### Different responses to immunotherapy of 3 ESCA metabolic subtypes

The TIDE algorithm is a useful tool for modeling two major tumor immune infiltration mechanisms: preventing T-cell infiltration with low cytotoxic T-lymphocyte (CTL) levels and stimulating T-cell dysfunction with high CTL levels. In this study, the TIDE algorithm was employed to investigate whether there are significant differences in the response to immunotherapy among the different subtypes in the GEO and TCGA-ESCA datasets.

The results indicated that the TIDE score of the MC3 subtype in the TCGA cohort was significantly higher compared to the MC1 and MC2 subtypes (Supplementary Fig. 2A-B). Additionally, the percentage of immunotherapy responders was significantly lower in the MC3 subtype, suggesting a higher probability of immune escape and a lower likelihood of benefiting from immunotherapy.

To further validate these findings, the subclass mapping method was used to compare the similarity of our defined three metabolic subtypes to patients in the GSE7220 dataset with different responses to immunotherapy. Lower p-values indicate higher similarity. The analysis revealed that the MC3 subtype was insensitive to PD-1 inhibitors (Supplementary Fig. 2E). Similar results were obtained when analyzing the GSE19417 dataset (Supplementary Fig. 2C, D and F). This indicate that samples belonging to the MC3 subtype exhibited the poorest response to immunotherapy, while those in the MC1 subtype showed the highest sensitivity to immunotherapy.

Furthermore, the study investigated the differences in the sensitivity of different subtype samples in the TCGA-ESCA dataset to chemotherapeutics. The analysis revealed that the MC2 subtype was more sensitive to gemcitabine, cisplatin, and docetaxel, whereas the MC3 subtype was more sensitive to paclitaxel than the other subtypes (Supplementary Fig. 2G).

### The construction of a typical metabolic index

In this study, a typical indicator model for ESCA sample classification was constructed using the Latent Dirichlet Allocation (LDA) model. The different colors in Supplementary Fig. 3A represent the diverse metabolic subtypes, showing concentrated distributions and distinct inter-category distances.

Significant differences in LDA scores were observed among the three TCGA subgroups, with higher scores in the MC2 and MC3 subgroups and lower scores in the MC1 subgroup (Supplementary Fig. 2B). Similar significant differences in LDA scores were observed among the diverse subgroups from the GEO databases, with significantly higher scores in the MC3 subgroup and lower scores in the MC1 and MC2 subgroups (Supplementary Fig. 3C and D). The consistency of LDA scores between the two databases suggests the stability of the metabolic subtypes across diverse datasets.

Furthermore, receiver operating characteristic (ROC) curves were generated to assess the accuracy of the typical index in predicting prognosis in the TCGA dataset, with an area-under-the-curve (AUC) value of 0.87 (Supplementary Fig. 3E). Similarly, for the GEO dataset, the multiclass AUC value was determined to be 0.96 (Supplementary Fig. 3F). These multiclass AUC values indicate that the typical metrics used to measure different metabolic characteristics demonstrate favorable accuracy in classifying ESCA cases.

### Metabolic indices associated with co-expressed genes

**Fig. 4:**
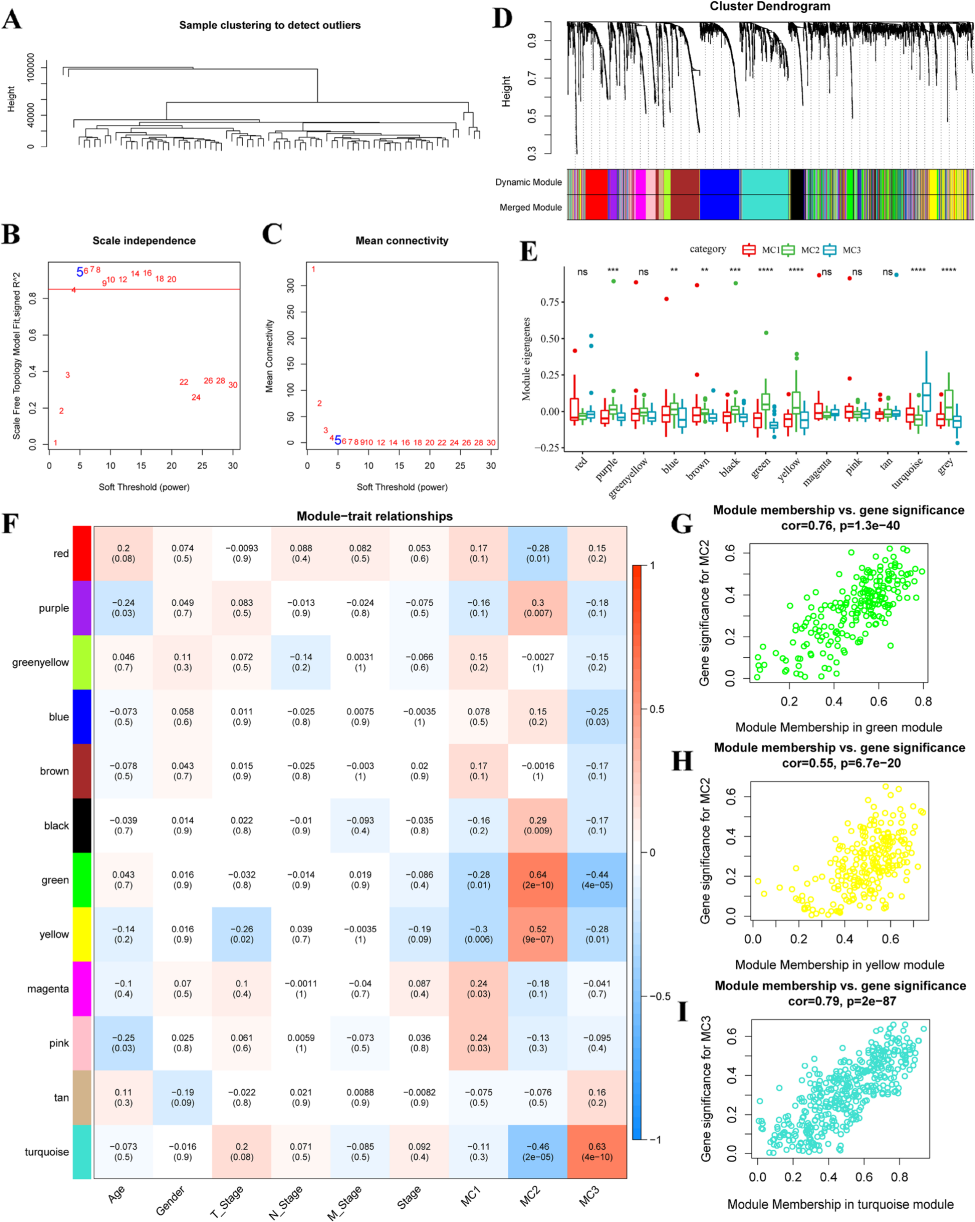
Weighted gene co-expression network analysis (WGCNA) of metabolic subtype characteristic index-related genes in the three ESCA subtypes in the TCGA cohort. Analysis of network topology for various soft thresholding powers (**A**-**C**). **D** Hierarchical cluster tree displaying 12 modules of co-expressed genes. **E** The distribution of eigenvectors of 13 modules in the 3 metabolic subtypes. **F** Heatmap showing the correlations of each module with Age, Gender, T Stage, N Stage, M Stage, MC1, MC2 and MC3 subtypes. **G** Scatter diagram for module membership vs. gene significance for MC2 in the green module. **H** Scatter diagram for module membership vs. gene significance for MC2 in the yellow module. **I** Scatter diagram for module membership vs. gene significance for MC3 in the turquoise module

The gene module was determined using the dynamic shear method, and the corresponding eigenvector values were calculated. Clustering analysis was performed on all modules, and closed modules were merged into a new module using the parameters: depth split = 1, minimum module size = 30, and height = 0.3. A total of 13 modules were obtained (Fig. 4A-D). The distribution of eigenvectors for these 13 modules in the three metabolic subtypes was calculated (Fig. 4E). Eight out of the 13 modules showed significant differences in their distribution among the three molecular subtypes. Notably, the green and yellow modules were significantly associated with the MC2 subtype, while the turquoise module was significantly enriched in the MC3 subtype.

**Fig. 5:**
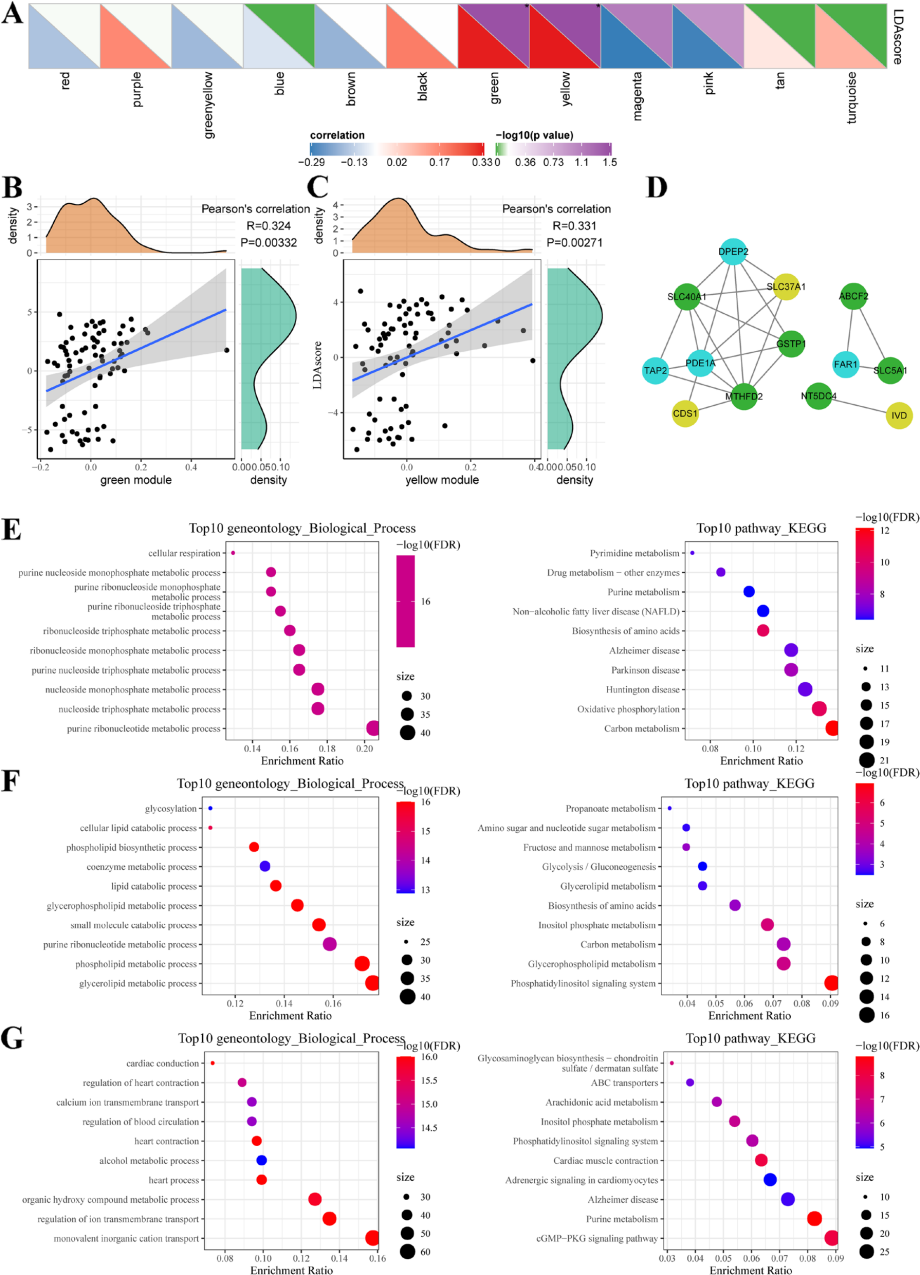
Mining hub gene-related metabolic subtype characteristic indices. **A** Correlation analysis of module feature vector and metabolic subtype characteristic index. **B** The relationship between the eigenvector of the green module and the characteristic index. **C** The relationship between the eigenvector of the yellow module and the characteristic index. **D** Co-expression network of hub genes associated with metabolic subtype characteristic index. **E** GO-BP and KEGG enrichment analysis of genes in the green module. **F** GO-BP and KEGG enrichment analysis of genes in the yellow module. **G** GO-BP and KEGG enrichment analysis of genes in the turquoise module

Correlation analysis was conducted between the feature vectors of each module and age, sex, T-stage, N-stage, M-stage, and the MC1, MC2, and MC3 subtypes (Fig. 4F-I). The green and yellow modules showed significant positive correlations with the MC2 subtype, while the turquoise module displayed a significant positive correlation with the MC3 subtype. Additionally, the eigenvectors of the green and yellow modules exhibited significant positive correlations with the constructed typical metabolic index (Fig. 5A-C).

Enrichment analysis of the modules (Fig. 5E-G) revealed that the green module is associated with nucleic acid metabolism and oxidative phosphorylation processes. The yellow module is related to glycolipid metabolism processes, such as cellular lipid catabolism, phospholipid biosynthesis, and lipid catabolism. The genes in the turquoise module are primarily enriched in biological processes such as glycosaminoglycan synthesis, arachidonic acid metabolism, and protein transport.

Finally, to identify metabolism-related prognostic markers for ESCA patients, 13 genes (Fig. 5D) with a module co-expression weight > 0.5 and significant correlation with the typical metabolic index were selected from the green, yellow, and turquoise modules. Among them, six genes (SLC40A1, GSTP1, MTHFD2, NT5DC4, ABCF2, and SLC5A1) were derived from the green module, three genes (CDS1, SLC37A1, and IVD) from the yellow module and four genes (DPEP2, TAP2, PDE1A, and FAR1) from the turquoise module. The correlation between gene expression and patient outcomes was analyzed by classifying patients into high and low expression groups based on the expression levels of these 13 genes. The results (Supplementary Fig. 4) indicated that only three genes (SLC5A1, NT5DC4, and MTHFD2) from the green module were significantly associated with prognosis.

### Expression levels of MTHFD2, SLC5A1 and NT5DC4 in ESCA

**Fig. 6:**
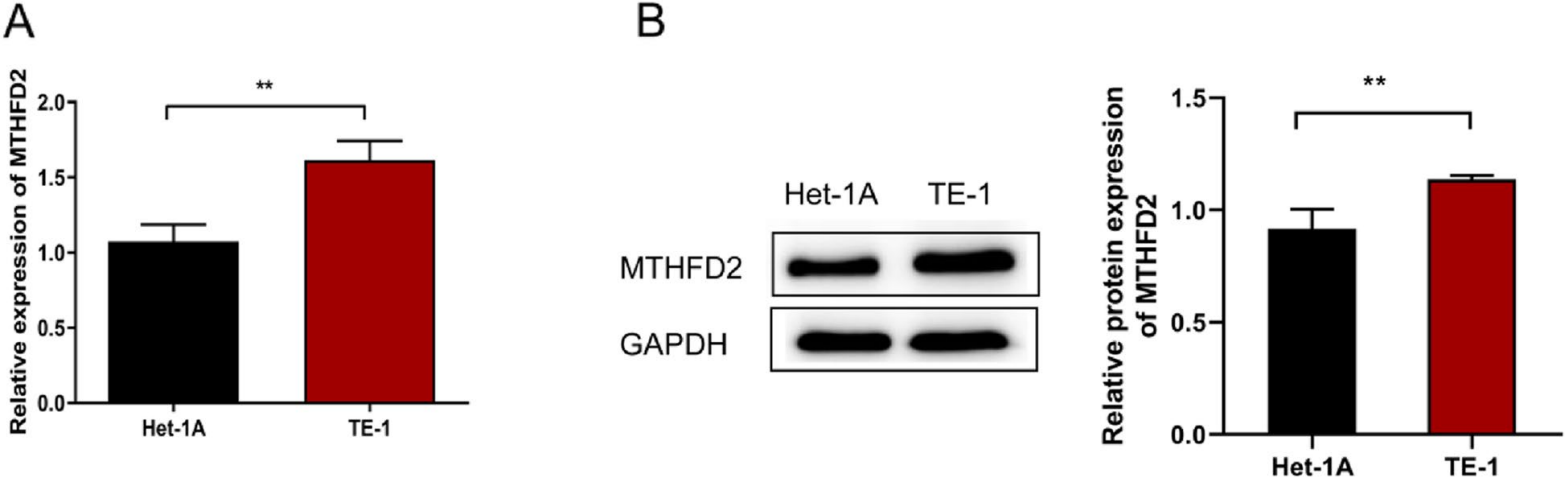
The expression of MTHFD2 is up-regulated in ESCA. The expression level of MTHFD2 in ESCA cell lines was detected by qRT-PCR (**A**) and western blot (**B**). **p < 0.01

Based on the obtained results above, ESCA tissue and adjacent normal tissues were retrieved from ESCA patients perioperatively, and the expression levels of the three promising genes were assessed, which were then further validated in ESCA cell lines. qRT-PCR analysis revealed distinct gene expression patterns in different comparisons. In the ESCA group, the MTHFD2 expression was found to be significantly increased, while SLC5A1 and NT5DC4 showed a slight but nonsignificant increase compared to the Adjacent tissue group (Supplementary Fig. 5A-C). In the cell lines, TE-1 cells exhibited significantly higher MTHFD2 expression compared to the Het-1A group, indicating upregulation in TE-1 cells (Fig. 6A), which was validated in western blot experiments (Fig. 6B). These findings suggest that MTHFD2 may be a potential target for ESCA and are worth further exploration.

### MTHFD2 knockdown inhibited the proliferation and invasion of ESCA cells

**Fig. 7:**
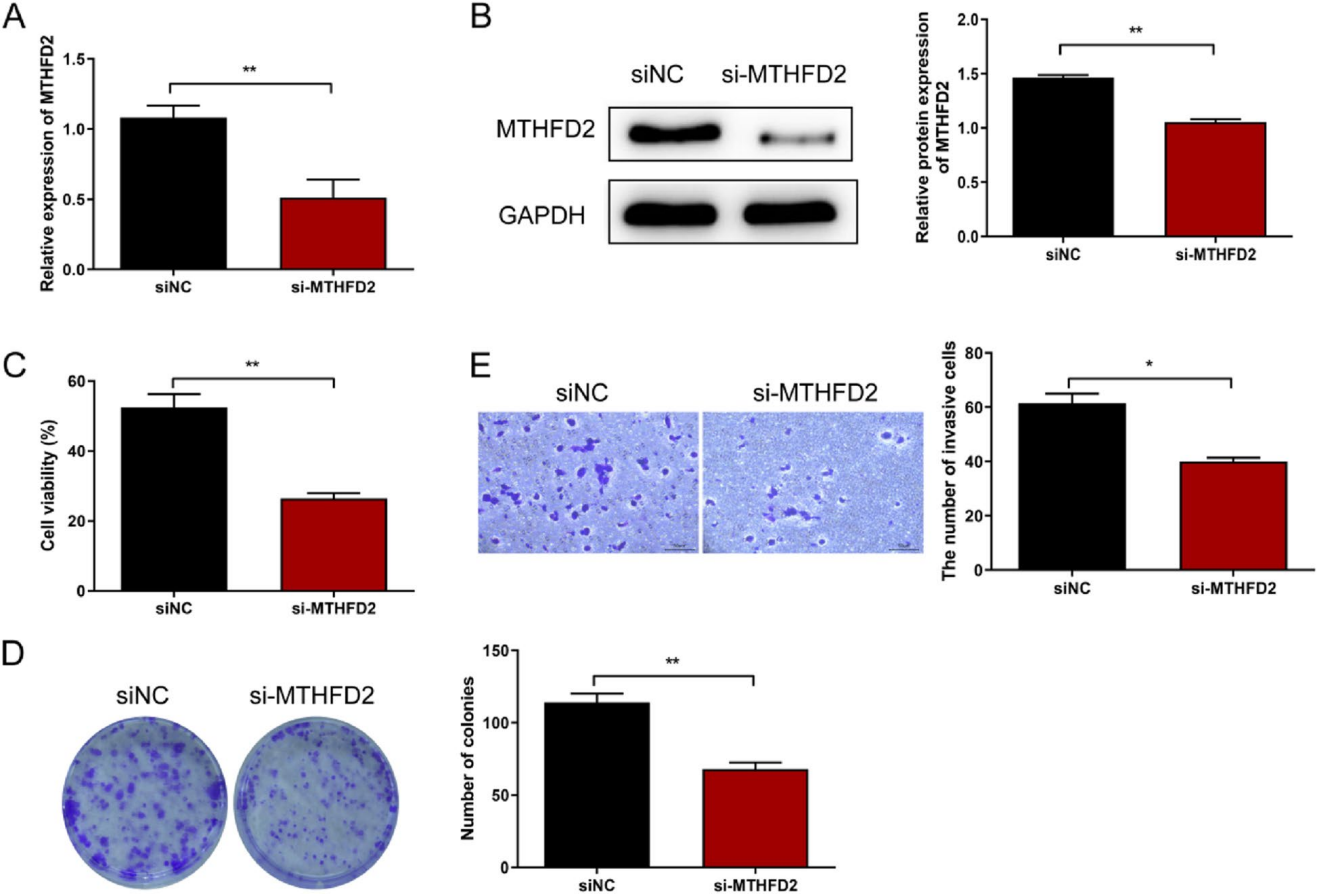
MTHFD2 knockdown inhibited the proliferation and invasion of ESCA cells. **A** qRT-PCR assay to evaluate the transfection effect of MTHFD2 in TE-1 cells. **B** Western blot assay confirmed the transfection efficiency of MTHFD2 in TE-1 cells. **C**-**E** Functional experiments demonstrating the effects of downregulating MTHFD2 on (**C**) cell viability via CCK-8 assay, (**D**) the ability of ESCA cells to form colonies via clonogenic assay, and (**E**) promoting ESCA cell invasion via Transwell assay. **p < 0.01

In order to explore the effect of MTHFD2 on ESCA, we conducted transfection experiments to knock down the expression of MTHFD2. The results showed that after transfection, the expression of MTHFD2 in the cells of the si-MTHFD2 knocking group was significantly reduced compared with that in the siNC group (Fig. 7A). The transfection efficiency was further verified by Western blot analysis (Fig. 7B).

Subsequently, the viability of si-MTHFD2 group cells was assessed using the CCK-8 assay to evaluate the impact of MTHFD2 downregulation on cell survival and proliferation. The results demonstrated a significant decrease in cell viability in the si-MTHFD2 group compared to the siNC group (Fig. 7C). The reduced viability of si-MTHFD2 group cells suggests the involvement of MTHFD2 in promoting cell growth and survival in ESCA.

To investigate the effect of MTHFD2 downregulation on cell proliferation and clonogenic potential, the colony formation assay was conducted. The results revealed a significant reduction in the number of cell colonies in the si-MTHFD2 group compared to the siNC group (Fig. 7D). The results indicate that MTHFD2 downregulation impairs the ability of ESCA cells to form colonies.

In addition, the invasive ability of si-MTHFD2 group cells was evaluated using the Transwell assay, which assesses cell migration and invasion. The results showed a significant decrease in the invasive ability of si-MTHFD2 group cells compared to the siNC group. This reduced invasiveness of si-MTHFD2 group cells suggests that MTHFD2 plays a crucial role in promoting cell invasion in ESCA (Fig. 7E). Overall, these functional assays provide valuable insights into the functional role of MTHFD2 and highlight its potential as a promising therapeutic target in ESCA.

## Discussion

Metabolic reprogramming is a well-established hallmark in cancer [36], closely related to ESCA proliferation, migration, and invasion by interacting with immune cells, stromal cells, as well as immune cell interactions and stromal crosstalk [37, 38]. It has been suggested that metabolic reprogramming reduces immune responses by starving T cells and secreting suppressive metabolites [39, 40]. A pan-cancer study revealed that the expression profiles of genes associated with the metabolic pathway may indicate the actual metabolic activity of patients [41]. Therefore, it is important to identify metabolic subtypes and associated immune landscapes to discover metabolic heterogeneities and analyze survival differences in ESCA cases. Additionally, constructing metabolic characteristic indicators can help evaluate and stratify patient outcomes and provide novel targets for treatment strategies.

In this study, we used bioinformatics to identify three metabolic subtypes (MC1-MC3) with different metabolic profiles. Among the subtypes, MC3 showed an increased proportion of immune cells and immune pathways, along with improved prognosis. However, further analyses suggest that MC3 increases the TIDE score, indicating a decrease in response to immunotherapy, and these findings were validated as signatures of immunosuppression. The MC3 subtype had increased stromal scores, which have been related to tumor development through the remodeling of anticancer immunity and immunotherapy response [42, 43]. Overall, combining immunotherapy with anti-metabolites targeting metabolic pathways, such as glycosaminoglycan metabolism, may suppress immune dysfunction in MC3 subtype patients. On the other hand, the MC1 subtype samples had the poorest prognosis, with the second-highest immunosuppression score and immune cell infiltration level and the lowest TIDE score but the strongest response to immunotherapy. These results suggest that the tumor microenvironment in this subtype exhibits distinct immune activation status, emphasize the need for tailored treatment strategies and highlight the potential of combining metabolic and immunotherapeutic approaches for ESCA patients.

The present study employed WGCNA and the LDA algorithm to identify key metabolic genes across the three ESCA sample subtypes using a typical characteristic index. The results revealed that the genes associated with the typical index were clustered into 13 distinct modules. Notably, the yellow, green, and turquoise modules exhibited significant correlations with the typical metabolic indices. From these modules, 13 characteristic genes (SLC40A1, GSTP1, MTHFD2, NT5DC4, ABCF2, SLC5A1, CDS1, SLC37A1, IVD, DPEP2, TAP2, PDE1A, and FAR1) were selected as key metabolic genes for ESCA cases, their potential in predicting patient prognosis was assessed, and the results indicated that SLC5A1, NT5DC4 and MTHFD2 showed significant associations with ESCA prognosis. The robustness and reproducibility of the metabolic index across diverse datasets enhance its clinical potential for assisting in prognosis assessment and guiding personalized treatment decisions.

Two of the three genes mentioned above have been implicated in cancer-related processes. SLC5A1, also known as sodium/glucose transporter 1 (SGLT1), is highly expressed in various tumors and contributes to the uptake of glucose by tumor cells, supporting their glycolytic metabolism. It has been associated with cancer cell growth, metastasis, and poor survival outcomes [44]. Methylenetetrahydrofolate dehydrogenase 2 (MTHFD2) is a key enzyme involved in the m1C cycle, which plays a role in metabolic reprogramming, immune evasion, and disease progression in multiple cancers, including prostate cancer, lung adenocarcinoma, and ESCA [45–49]. However, the association of NT5DC4 with patient survival has not been previously reported, and its specific role in regulating the biological functions of tumor cells needs to be further investigated in in vivo and in vitro studies.

The gene expression analysis conducted in ESCA samples and cell lines revealed distinct expression patterns of the three promising genes (MTHFD2, SLC5A1, and NT5DC4) compared to normal tissues and control cell lines. Specifically, MTHFD2 expression was significantly increased in ESCA samples, indicating its upregulation in the disease context, suggesting its potential involvement in promoting tumor growth and progression. The subsequent protein expression analysis validated the gene expression findings of MTHFD2, reinforcing its relevance in ESCA and demonstrating its potential as a biomarker for the disease. To evaluate the functional impact of MTHFD2 downregulation, functional assays were performed. Overall, the functional characterization assays provide important insights into the functional role of MTHFD2 in ESCA, demonstrating its involvement in cell viability, proliferation, colony formation and invasion. These findings are in light with previous literature [50], support the significance of MTHFD2 as a potential therapeutic target in ESCA and suggest that targeting MTHFD2 could be a promising approach to inhibit tumor growth and progression, as well as a link between the identified metabolic subtypes and their associated molecular mechanisms, adding to the overall understanding of ESCA heterogeneity and its clinical implications.

Overall, the findings of this study have potential clinical impacts. The identification of metabolic subtypes and their associated metabolic profiles can aid in refining prognosis assessment and personalizing treatment strategies for ESCA patients [51]. The differential immune cell infiltration observed among the subtypes suggests the potential for tailored immunotherapeutic approaches. The construction of a metabolic index with high accuracy in classifying ESCA cases can assist in clinical decision-making and contribute to precision medicine [52]. Moreover, the identification of prognostic metabolic genes, such as MTHFD2, provides potential targets for therapeutic interventions and opens avenues for developing novel treatment strategies. To further advance the field, future studies could explore the functional mechanisms through which the identified metabolic genes, including MTHFD2, contribute to ESCA progression and treatment response. Additionally, investigations into the crosstalk between metabolic reprogramming and immune modulation within the tumor microenvironment could provide deeper insights into the interplay between metabolism and the immune system in ESCA. Longitudinal studies involving larger patient cohorts and prospective clinical trials are needed to validate the clinical utility of the identified metabolic subtypes, metabolic index, and prognostic genes, ultimately translating these findings into improved patient outcomes and more effective therapeutic interventions for ESCA.

Despite the interesting findings reported, there were several limitations that should be acknowledged. Firstly, although the inclusion of 150 cases from RNA-seq and microarray platforms enhances the reliability and robustness of the findings, prospective studies are necessary to validate whether the identified key metabolic genes can serve as prognostic indicators and predictors of response to immunotherapy in patients with ESCA. Secondly, this study relied on bioinformatic analysis of publicly accessible cancer databases, and further studies involving clinical samples are warranted to validate the identified hallmarks. Thirdly, additional investigations are needed to elucidate the underlying mechanisms linking metabolic modulation and ESCA prognosis for the identified metabolic genes. Lastly, exploring whether intratumoral metabolic features undergo changes following treatment in ESCA patients and whether such changes influence patient response to immunotherapy is important.

## Conclusion

In conclusion, this study successfully identified three distinct metabolic subtypes in ESCA and characterized the immune networks and metabolic pathways associated with each subtype. The findings enhance our understanding of the interplay between tumor metabolism and immunity and suggest the potential benefits of combining immunotherapy with anti-metabolite treatments to enhance anticancer immunity. Additionally, the identified metabolic genes MTHFD2 exhibited strong predictive ability, and may be potential therapeutic targets for ESCA. Overall, these findings contribute to our knowledge of ESCA heterogeneity and provide potential targets for personalized therapeutic approaches.

### Supplementary Information

**Additional file 1: Supplementary Table 1. **Clinicopathologic features of ESCA patients in this study. **Supplementary Table 2. **Primer sequence.

**Additional file 2: Supplementary Figure 1. **Clinical characteristics of samples from different subtypes in the TCGA and GEO cohorts. (A) T Staging ratio distribution in the three ESCA subtypes in TCGA-ESCA cohort. (B) N Staging ratio distribution in the three ESCA subtypes in TCGA-ESCA cohort. (C) M Staging ratio distribution in the three ESCA subtypes in TCGA-ESCA cohort. (D) Tumor stage ratio distribution in the three ESCA subtypes in the TCGA-ESCA cohort. (E) Age distribution in the three ESCA subtypes in the TCGA-ESCA cohort. (F) Gender ratio distribution in the three ESCA subtypes in the TCGA-ESCA cohort. (G) Grade ratio distribution in the three ESCA subtypes in the GEO-ESCA cohort. (H) Gender ratio distribution in the three ESCA subtypes in the GEO-ESCA cohort. **Supplementary Figure 2.** Different responses to immunotherapy for 3 ESCA metabolic subtypes. (A) Differences in TIDE scores among 3 subtypes of samples from the TCGA-ESCA cohort. (B) Differences in response status to immunotherapy among 3 subtypes of samples from TCGA-ESCA cohort. (C) Differences in TIDE scores among 3 subtypes of samples from the GEO-ESCA cohort. (D) Differences in response status to immunotherapy among 3 subtypes of samples from the GEO-ESCA cohort. (E) Submap analysis showed that MC3 was not sensitive to the PD-1 inhibitor (Bonferroni-corrected *P* < 0.05) in the TCGA cohort. (F) Submap analysis manifested that MC1 could be sensitive to the PD-1 inhibitor in the GSE19417 cohort. (G) The box plots of the estimated IC50 for gemcitabine, Cisplatin, paclitaxel and docetaxel on samples in 3 subtypes from the TCGA-ESCA cohort. **Supplementary Figure 3. **Construction of the metabolic subtype characteristic index model. (A) The first 2 characteristics of the model were able to distinctly classify the TCGA-ESCA samples into 3 different subtypes. (B) The characteristic index of samples in three ESCA subtypes from the TCGA cohort. (C) The ROC curve of metabolic subtype characteristic index in TCGA-ESCA cohort. (D) The first 2 characteristics of the model were able to distinctly classify the GEO-ESCA samples into 3 different subtypes. (E) The characteristic index of samples in three ESCA subtypes from the GEO cohort. (F) The ROC curve of metabolic subtype characteristic index in GEO-ESCA cohort. **Supplementary Figure 4.** Kaplan–Meier curves showing the distinct outcomes of ESCA patients with different expression levels of SLC40A1, GSTP1, MTHFD2, NT5DC4, ABCF2, SLC5A, CDS1, SLC37A1, IVD, DPEP2, TAP2, PDE1A and FAR1. **Supplementary Figure 5.** Expression levels of MTHFD2, SLC5A1 and NT5DC4 in ESCA. qRT-PCR analysis for validating the expresion of (A) MTHFD2, (B) SLC5A1 and (C) NT5DC4 in ESCA. **p < 0.01.

**Additional file 3: Figure 6B.** The original Western Blot images of MTHFD2 in Figure 6B. From left to right: Het-1A, TE-1. The original Western Blot images of GAPDH in Figure 6B. From left to right: Het-1A, TE-1. **Figure 7B.** The original Western Blot images of MTHFD2 in Figure 7B. From left to right: siNC, si-MTHFD2. The original Western Blot images of GAPDH in Figure 7B. From left to right: siNC, si-MTHFD2.

## Data Availability

The authors confirm that the data supporting the findings of this study are available within the article.
