# Supplementary material for: Metabolic subtypes and immune landscapes in esophageal squamous cell carcinoma: prognostic implications and potential for personalized therapies
Source: BMC Cancer. 2024 Feb 19;24:230. doi: 10.1186/s12885-024-11890-x (PMC10875771; doi:10.1186/s12885-024-11890-x)
Supplement: Supplementary file 1 — Additional file 1: Supplementary Table 1. Clinicopathologic features of ESCA patients in this study. Supplementary Table 2. Primer sequence. [file 12885_2024_11890_MOESM1_ESM.docx]

**Supplementary Table 1** Clinicopathologic features of ESCA patients in this study.

| Features | Esophageal carcinoma (n = 27) |
| --- | --- |
| Age, years (mean) | 63.61 |
| Gender |  |
| Male | 18 |
| Female | 9 |
| Stage |  |
| T2N1 | 4 |
| T3N0 | 4 |
| T3N1 | 11 |
| T3N2 | 5 |
| T3N3 | 4 |

**Supplementary Table 2** Primer sequence.

| Gene name | Primer sequences (5’ to 3’) |
| --- | --- |
| SLC5A1 | F: AAAATTGCCTGTGTCGTCCC |
|  | R: TTGGCGTAGATGTCCATGGT |
| NT5DC4 | F: ACCTGCCTGAAACCTACCTC |
|  | R: TCCTTCATCTTCCCCAGCAG |
| MTHFD2 | F: CCTGGCTATCTATCCACCATGTG |
|  | R: TTCTGGTCCTCGTCTTGCCTGT |
| TEAD4 | F: GGGAAGAATGTGGTTGTGGC |
|  | R: ATGACTGCTGCTCCTTCCTT |
| GAPDH | F: GTCTCCTCTGACTTCAACAGCG |
|  | R: ACCACCCTGTTGCTGTAGCCAA |
