# Supplementary material for: Metabolic subtypes and immune landscapes in esophageal squamous cell carcinoma: prognostic implications and potential for personalized therapies
Source: BMC Cancer. 2024 Feb 19;24:230. doi: 10.1186/s12885-024-11890-x (PMC10875771; doi:10.1186/s12885-024-11890-x)
Supplement: Supplementary file 2 — Additional file 2: Supplementary Figure 1. Clinical characteristics of samples from different subtypes in the TCGA and GEO cohorts. (A) T Staging ratio distribution in the three ESCA subtypes in TCGA-ESCA cohort. (B) N Staging ratio distribution in the three ESCA subtypes in TCGA-ESCA cohort. (C) M Staging ratio distribution in the three ESCA subtypes in TCGA-ESCA cohort. (D) Tumor stage ratio distribution in the three ESCA subtypes in the TCGA-ESCA cohort. (E) Age distribution in the three ESCA subtypes in the TCGA-ESCA cohort. (F) Gender ratio distribution in the three ESCA subtypes in the TCGA-ESCA cohort. (G) Grade ratio distribution in the three ESCA subtypes in the GEO-ESCA cohort. (H) Gender ratio distribution in the three ESCA subtypes in the GEO-ESCA cohort. Supplementary Figure 2. Different responses to immunotherapy for 3 ESCA metabolic subtypes. (A) Differences in TIDE scores among 3 subtypes of samples from the TCGA-ESCA cohort. (B) Differences in response status to immunotherapy among 3 subtypes of samples from TCGA-ESCA cohort. (C) Differences in TIDE scores among 3 subtypes of samples from the GEO-ESCA cohort. (D) Differences in response status to immunotherapy among 3 subtypes of samples from the GEO-ESCA cohort. (E) Submap analysis showed that MC3 was not sensitive to the PD-1 inhibitor (Bonferroni-corrected P < 0.05) in the TCGA cohort. (F) Submap analysis manifested that MC1 could be sensitive to the PD-1 inhibitor in the GSE19417 cohort. (G) The box plots of the estimated IC50 for gemcitabine, Cisplatin, paclitaxel and docetaxel on samples in 3 subtypes from the TCGA-ESCA cohort. Supplementary Figure 3. Construction of the metabolic subtype characteristic index model. (A) The first 2 characteristics of the model were able to distinctly classify the TCGA-ESCA samples into 3 different subtypes. (B) The characteristic index of samples in three ESCA subtypes from the TCGA cohort. (C) The ROC curve of metabolic subtype characteristic index in TCGA-ES [file 12885_2024_11890_MOESM2_ESM.docx]

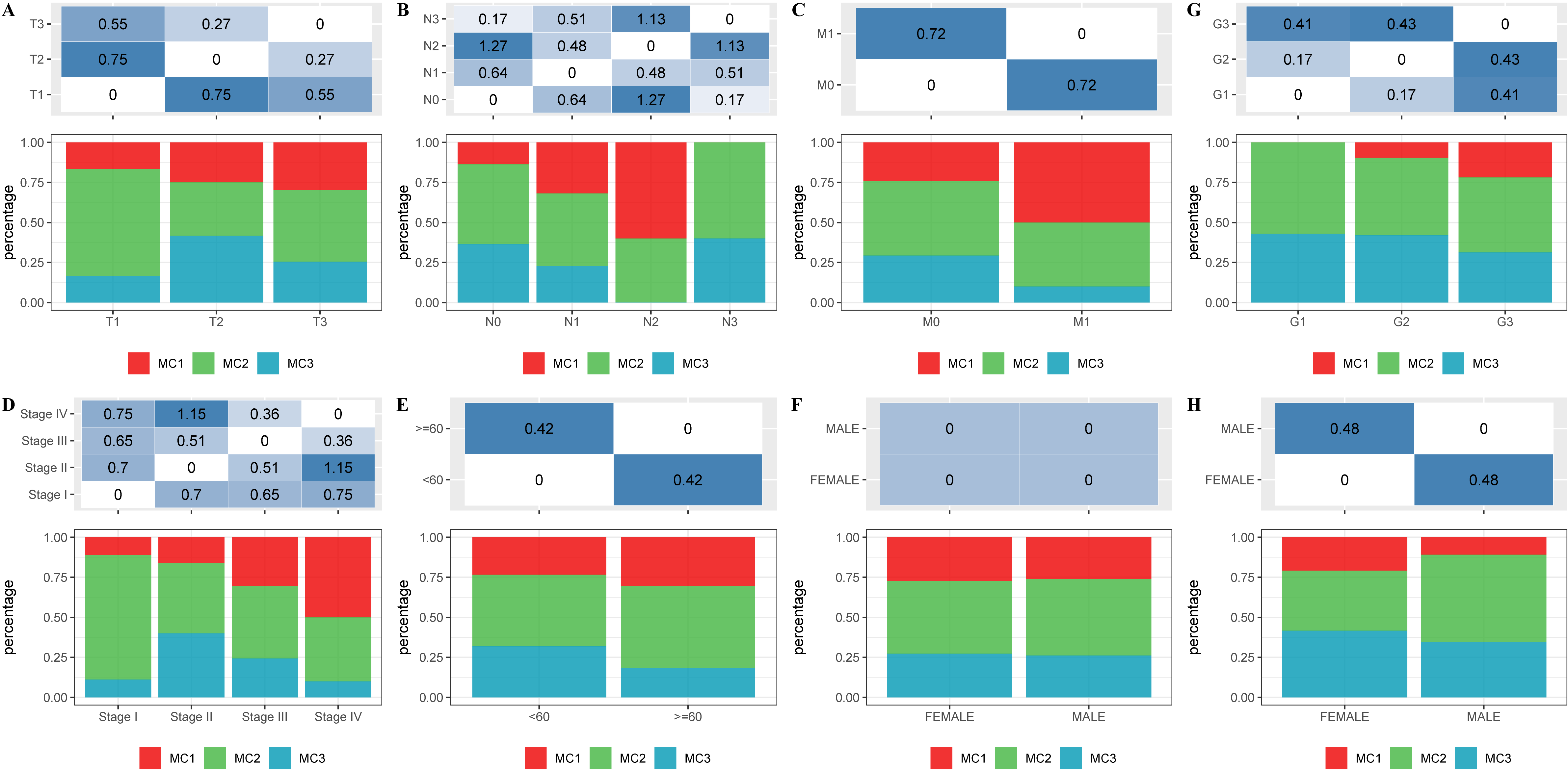


**Supplementary Figure 1** Clinical characteristics of samples from different subtypes in the TCGA and GEO cohorts. (A) T Staging ratio distribution in the three ESCA subtypes in TCGA-ESCA cohort. (B) N Staging ratio distribution in the three ESCA subtypes in TCGA-ESCA cohort. (C) M Staging ratio distribution in the three ESCA subtypes in TCGA-ESCA cohort. (D) Tumor stage ratio distribution in the three ESCA subtypes in the TCGA-ESCA cohort. (E) Age distribution in the three ESCA subtypes in the TCGA-ESCA cohort. (F) Gender ratio distribution in the three ESCA subtypes in the TCGA-ESCA cohort. (G) Grade ratio distribution in the three ESCA subtypes in the GEO-ESCA cohort. (H) Gender ratio distribution in the three ESCA subtypes in the GEO-ESCA cohort.


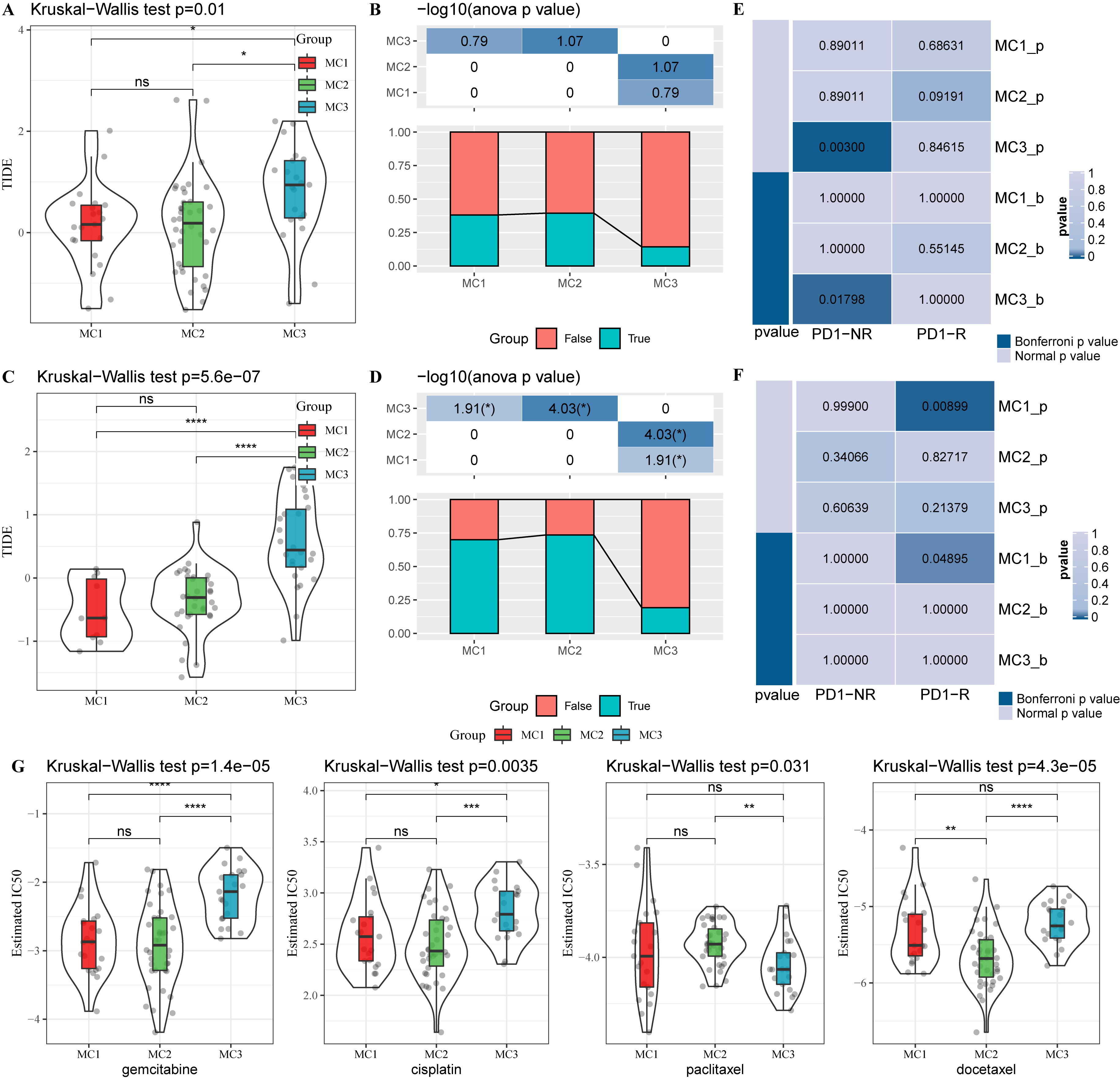


**Supplementary Figure 2** Different responses to immunotherapy for 3 ESCA metabolic subtypes. (A) Differences in TIDE scores among 3 subtypes of samples from the TCGA-ESCA cohort. (B) Differences in response status to immunotherapy among 3 subtypes of samples from TCGA-ESCA cohort. (C) Differences in TIDE scores among 3 subtypes of samples from the GEO-ESCA cohort. (D) Differences in response status to immunotherapy among 3 subtypes of samples from the GEO-ESCA cohort. (E) Submap analysis showed that MC3 was not sensitive to the PD-1 inhibitor (Bonferroni-corrected *P* < 0.05) in the TCGA cohort. (F) Submap analysis manifested that MC1 could be sensitive to the PD-1 inhibitor in the GSE19417 cohort. (G) The box plots of the estimated IC50 for gemcitabine, Cisplatin, paclitaxel and docetaxel on samples in 3 subtypes from the TCGA-ESCA cohort.


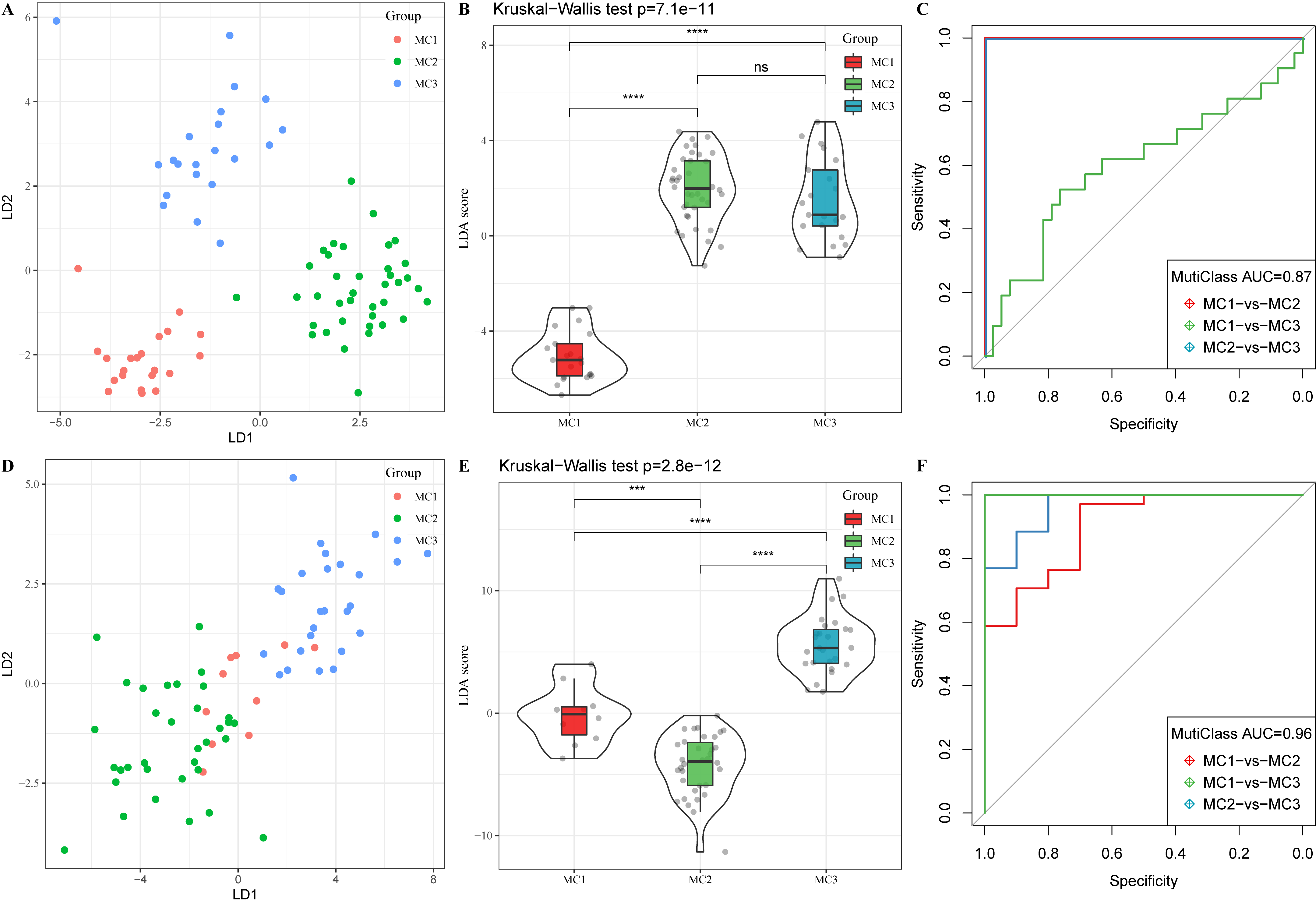


**Supplementary Figure 3** Construction of the metabolic subtype characteristic index model. (A) The first 2 characteristics of the model were able to distinctly classify the TCGA-ESCA samples into 3 different subtypes. (B) The characteristic index of samples in three ESCA subtypes from the TCGA cohort. (C) The ROC curve of metabolic subtype characteristic index in TCGA-ESCA cohort. (D) The first 2 characteristics of the model were able to distinctly classify the GEO-ESCA samples into 3 different subtypes. (E) The characteristic index of samples in three ESCA subtypes from the GEO cohort. (F) The ROC curve of metabolic subtype characteristic index in GEO-ESCA cohort.


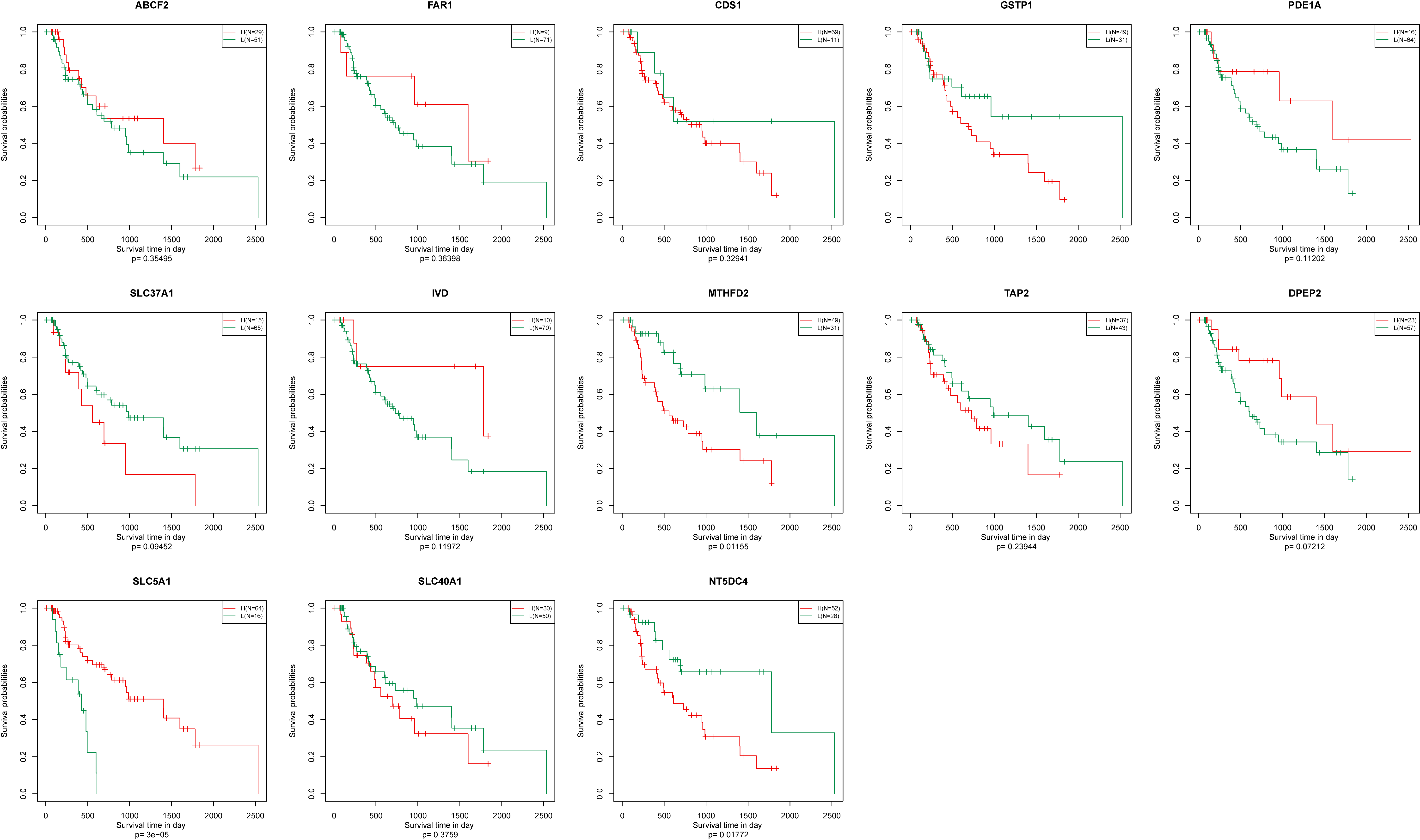


**Supplementary Figure 4** Kaplan–Meier curves showing the distinct outcomes of ESCA patients with different expression levels of SLC40A1, GSTP1, MTHFD2, NT5DC4, ABCF2, SLC5A, CDS1, SLC37A1, IVD, DPEP2, TAP2, PDE1A and FAR1.


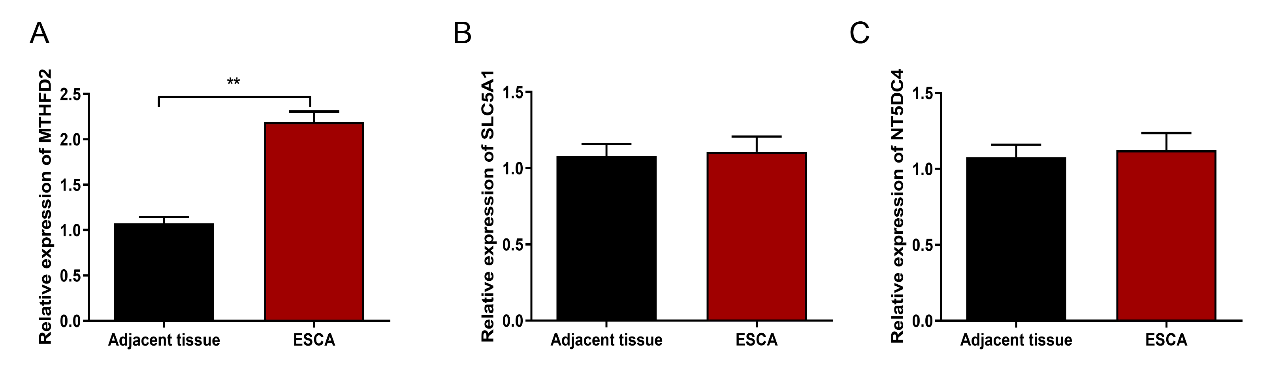


**Supplementary Figure 5.** Expression levels of MTHFD2, SLC5A1 and NT5DC4 in ESCA. qRT-PCR analysis for validating the expresion of (A) MTHFD2, (B) SLC5A1 and (C) NT5DC4 in ESCA. ***p* < 0.01.
