# Supplementary material for: Metabolic subtypes and immune landscapes in esophageal squamous cell carcinoma: prognostic implications and potential for personalized therapies
Source: BMC Cancer. 2024 Feb 19;24:230. doi: 10.1186/s12885-024-11890-x (PMC10875771; doi:10.1186/s12885-024-11890-x)
Supplement: Supplementary file 3 — Additional file 3: Figure 6B. The original Western Blot images of MTHFD2 in Figure 6B. From left to right: Het-1A, TE-1. The original Western Blot images of GAPDH in Figure 6B. From left to right: Het-1A, TE-1. Figure 7B. The original Western Blot images of MTHFD2 in Figure 7B. From left to right: siNC, si-MTHFD2. The original Western Blot images of GAPDH in Figure 7B. From left to right: siNC, si-MTHFD2. [file 12885_2024_11890_MOESM3_ESM.pdf]

Figure 6B

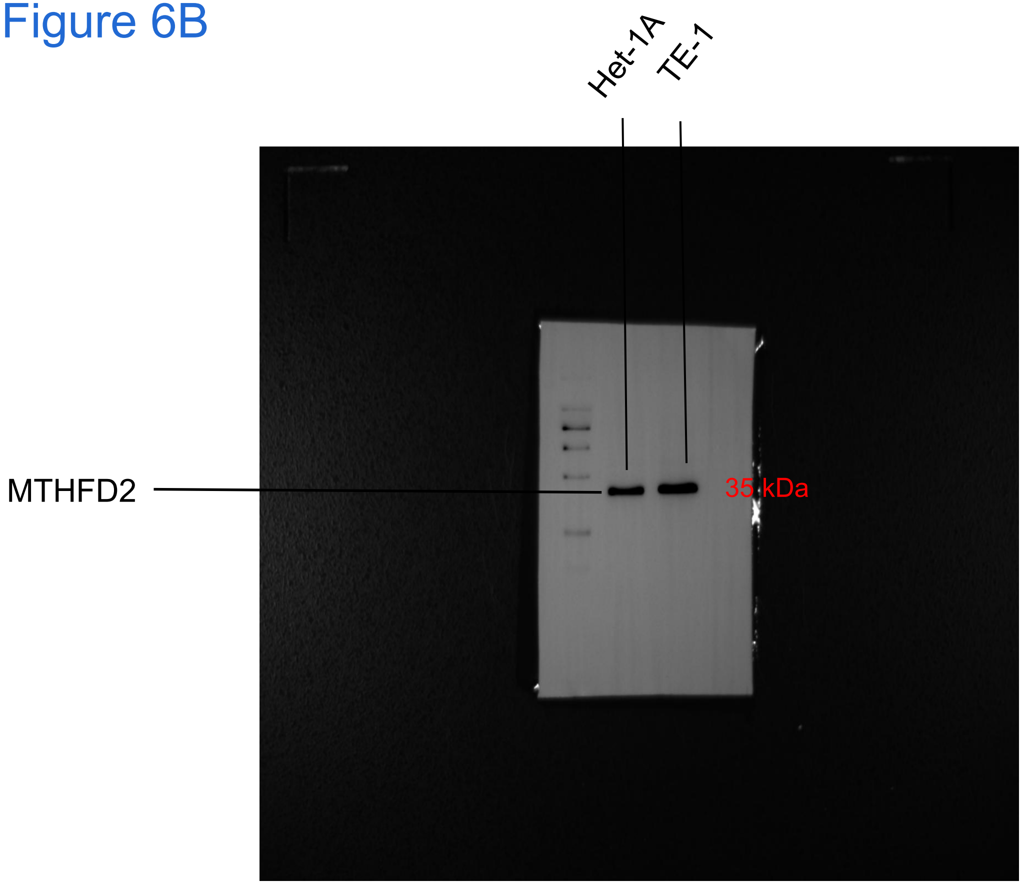

The original Western Blot images of MTHFD2 in Figure 6B. From left to right: Het-1A, TE-1.

Figure 6B

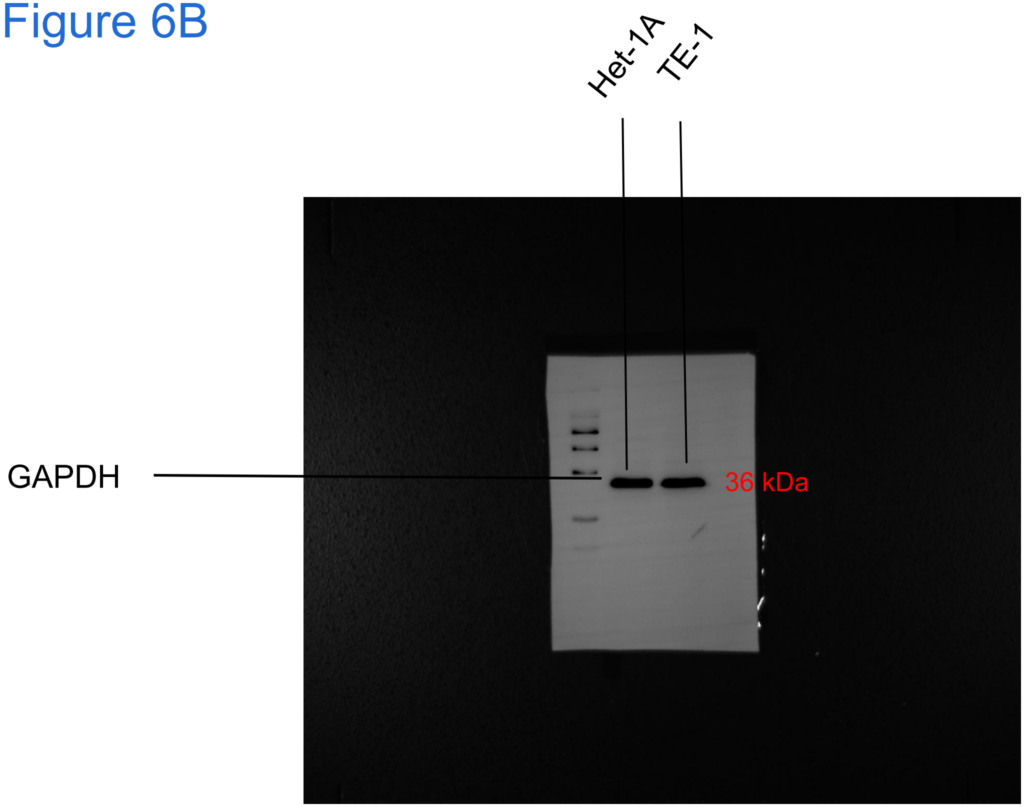

The original Western Blot images of GAPDH in Figure 6B. From left to right: Het-1A, TE-1.

Figure 7B

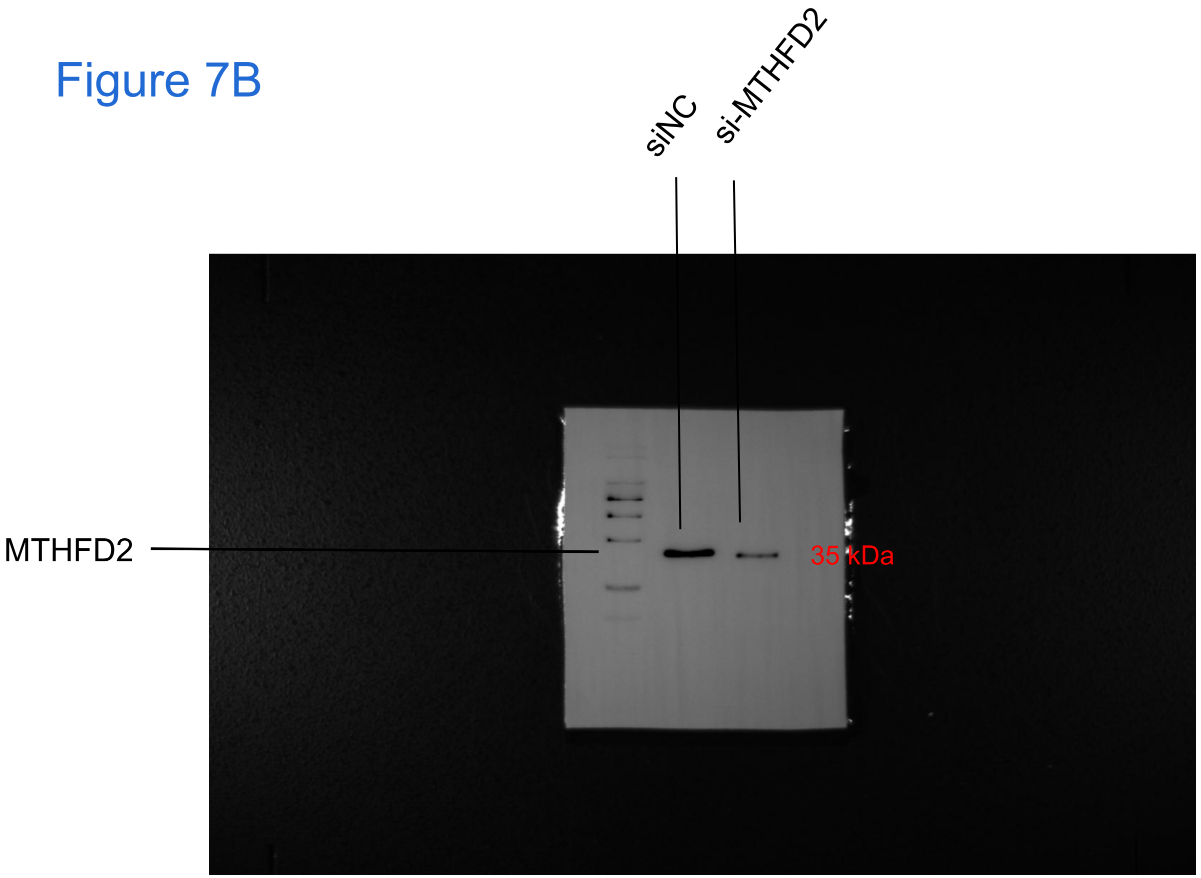

The original Western Blot images of MTHFD2 in Figure 7B. From left to right: siNC, si-MTHFD2.

Figure 7B

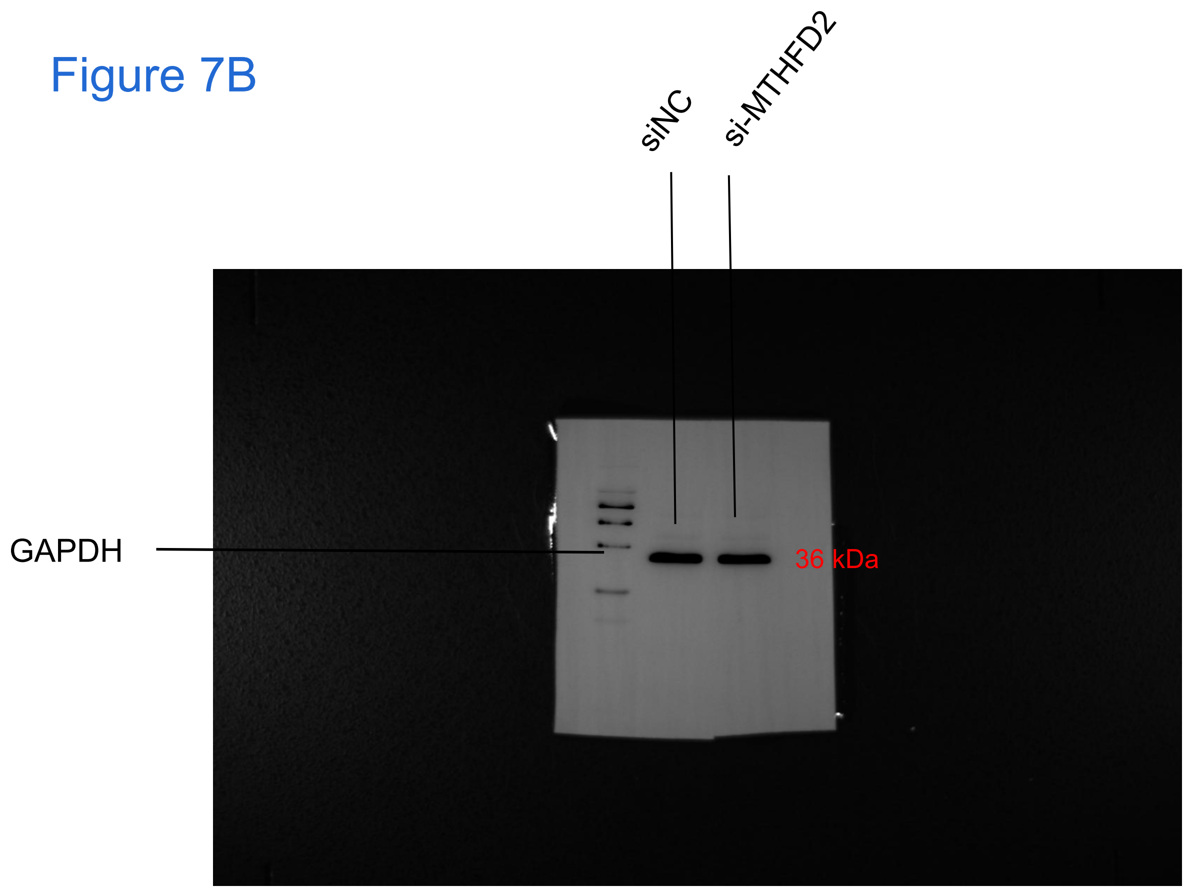

The original Western Blot images of GAPDH in Figure 7B. From left to right: siNC, si-MTHFD2.
